# Molecular and microenvironmental networks driving tumor dormancy: pathological mechanisms and emerging clinical strategies

**DOI:** 10.3389/fmed.2026.1979062

**Published:** 2026-09-29

**Authors:** Yuanshuai Li, Wenling Zhan, Min Min, Keyang Zhang, Yan Liu, Huang Chen, Xinlong Yan

**Affiliations:** 1 Beijing Key Laboratory of Environmental and Viral Oncology, College of Chemistry and Life Science, Beijing University of Technology, Beijing, China; 2 Department of Gastroenterology, First Medical Center of Chinese PLA General Hospital, Beijing, China; 3 Department of Pathology, China-Japan Friendship Hospital, Beijing, China

**Keywords:** relapse, therapy resistance, tumor dormancy, tumor microenvironment, tumor recurrence

## Abstract

Although standard therapies including surgery, radiotherapy, and chemotherapy have proven effective in the initial eradication of primary tumors, a substantial proportion of cancer patients remain at persistent threat of recurrence. Accumulating evidence identifies tumor dormancy as a pivotal driver of this clinical challenge. Dormant tumor cells can persist in a quiescent state for extended periods, during which they exhibit marked resistance to conventional therapies and may ultimately trigger disease relapse years after clinical remission. Consequently, deciphering the mechanisms governing tumor dormancy has become imperative for the development of novel therapeutic strategies aimed at preventing recurrence. Tumor dormancy arises from reciprocal interactions between tumor cells and their microenvironment, which collectively establishes a reservoir that fosters both drug tolerance and late relapse. This review systematically delineates the core molecular signatures, microenvironmental regulatory networks, and targeted intervention approaches relevant to dormant cancer cells, aiming to provide a theoretical foundation for the prevention of tumor recurrence.

## 1 Introduction

The post-therapeutic recurrence of solid tumors remains one of the most formidable challenges in clinical oncology, representing a major cause of treatment failure and cancer-related mortality (1, 2). Despite significant advances in primary therapies, such as surgery, radiotherapy, and chemotherapy, that have substantially prolonged patient survival, a substantial proportion of patients inevitably experience disease relapse (3–5). For instance, the postoperative recurrence rate for bladder cancer is high, ranging from 50% to 70%, underscoring the critical gap between initial tumor control and long-term cure (5–7). These latent lesions can remain asymptomatic for years or even decades following primary tumor resection, evading therapeutic intervention, until they are activated through mechanisms that are still poorly understood, ultimately culminating in fatal outgrowth (8, 9).

Tumor dormancy is the core biological process underlying post-therapeutic recurrence (10, 11). First described by Willis and later characterized by Hadfield as a reversible arrest of tumor cell proliferation (12), dormancy allows a subset of disseminated tumor cells (DTCs) to persist in distant organs, including bone marrow, lung, and liver (13–15), without forming overt tumor. These quiescent cells exhibit profound resistance to conventional therapies and can be reactivated by microenvironmental signals, ultimately driving late relapse (16–19).

Tumor dormancy is a critical stage in cancer progression and primarily comprises two forms: cellular dormancy and tumor mass dormancy (20). Cellular dormancy refers to a state in which individual tumor cells enter a slowly cycling or non-proliferative state characterized by reversible and temporary growth arrest. Tumor mass dormancy describes the existence of a tumor mass that achieves a dynamic equilibrium between cell proliferation and apoptosis, and is further subdivided into “angiogenic dormancy” and “immunogenic dormancy” (10, 20–22). As tumor masses expand, cancer cells depend on neovascularization for nutrient supply. Angiogenesis is finely regulated by pro-angiogenic factors and anti-angiogenic factors (23). Before the angiogenic switch, an increased number of cancer cells causes cell death in regions distant from pre-existing blood vessels due to a lack of oxygen and nutrients, thereby establishing an equilibrium between cancer cell proliferation and death, which is defined as angiogenic dormancy (21, 24). Immunogenic dormancy is a state of balanced cell proliferation and cell death based on surveillance by the host immune system (21, 25). Cancer cell proliferation and tumor growth can be suppressed by host immune surveillance, including T cells, B cells, and natural killer (NK) cells (26). For instance, effector T cells can kill tumor cells through cytokines such as IFN-*γ* and TNF-α. Meanwhile, if the host immune system fails to completely eliminate cancer cells, the remaining cancer cells may continue to proliferate (27, 28). Consequently, the tumor mass as a whole enters a proliferation–death equilibrium, resulting in immunogenic dormancy (21, 25).

Tumor dormant cell reactivation is frequently triggered by microenvironment remodeling induced by inflammation, tissue damage, or chemotherapy (29). For instance, pulmonary inflammation promotes the formation of neutrophil extracellular traps (NETs) that remodel the niche and awaken dormant cancer cells (30, 31). Similarly, chemotherapeutics such as doxorubicin and cisplatin can paradoxically enhance the metastatic potential of dormant breast cancer cells (32, 33). Thus, standard therapies, while effectively eliminating proliferating tumors, may inadvertently foster recurrence by altering the dormant cell microenvironment (34, 35). Accordingly, developing innovative strategies that either enforce permanent quiescence or selectively eliminate latent reservoirs represents a promising frontier for preventing recurrence and improving cure rates.

We systematically searched the PubMed (https://pubmed.ncbi.nlm.nih.gov/) and Web of Science (https://webofscience.clarivate.cn/wos/woscc/smart-search) databases up to August 2026, using the following keywords: “dormancy”, “tumor dormancy”, “cancer dormancy”, “disseminated tumor cells”, “dormancy awakening”, “tumor recurrence”, and “tumor metastases.” Only peer-reviewed original research articles, reviews, and clinical studies published in English were included. References were additionally identified by manual screening of the bibliographies of retrieved articles.

## 2 Molecular regulatory networks of dormant tumor cells

Tumor dormancy is a biological process cooperatively orchestrated by intracellular genetic, epigenetic, and post-transcriptional regulatory networks. Dormant tumor cells are capable of reactivation, which drives clinical relapse, and exhibit profound resistance to conventional cytotoxic agents. This section systematically delineates the core intracellular molecular networks governing tumor dormancy.

### 2.1 Molecular mechanisms of transcriptional regulation and cell cycle arrest

Cell cycle arrest is a central hallmark of the dormant state. Dormant tumor cells typically exit the active cell cycle and enter G_0_ phase, a non-proliferative state (19, 36). This process is cooperatively regulated by complex signaling pathways and intrinsic molecular networks. Key regulators include proteins known as cyclin-dependent kinase inhibitors, such as p21 and p27, which hinder the progression of the cell cycle and maintain cells in a dormant state (37, 38). Additionally, the DREAM (DP, RB, E2F, and MuvB) complex represses cell cycle-dependent genes during quiescence. DREAM contains p130, E2F4, DP1, and a stable MuvB core (LIN9, LIN37, LIN52, LIN54, and RBBP4) (38, 39). Upon cell cycle entry, the MuvB core dissociates from p130 and preferentially binds to BMYB to activate S-phase and G_2_/M-phase genes, thereby mediating the dynamic transcriptional switch between quiescence and proliferation (39).

The orphan nuclear receptor NR2F1 is recognized as a master transcriptional regulator of tumor dormancy (40). NR2F1 expression is markedly upregulated in dormant head and neck squamous cell carcinoma (HNSCC), as well as in DTCs from prostate cancer patients carrying dormant disease for 7 to 18 years. Combined treatment with the DNA demethylating agent 5-Aza-C and retinoic acid can induce NR2F1-dependent dormancy across multiple cancer types. NR2F1 drives cell cycle exit and entry into quiescence by upregulating SOX9, RAR*β*, and CDK inhibitors (41). Notably, NR2F1 also induces global chromatin repression while simultaneously activating the expression of the pluripotency factor NANOG, thereby contributing to the dormancy of DTCs in the bone marrow. When NR2F1 is blocked *in vivo*, growth arrest or survival of dormant DTCs is interrupted in different organs (40–42).

Beyond NR2F1, the maintenance of tumor cell dormancy relies on the coordinated activity of key signaling pathways. The ERK/p38 signaling ratio is considered as the core molecular switch that controls the dormancy-proliferation transition (43). A low ERK/p38 activity ratio promotes and sustains the dormant phenotype, a feature validated in clinical samples from various tumors, including fibrosarcoma and melanoma (18). Moreover, TGF*β*-2 in the microenvironment can bind its receptors, leading to a reduction in the intracellular ERK/p38 signaling ratio, which in turn upregulates the metastasis suppressor DEC2. DEC2 subsequently inhibits CDK4 and induces p27 expression, thereby establishing dormancy in bone marrow DTCs. Conversely, inhibiting p38 or blockade of TGF*β*-2 receptors disrupt this balance, reactivating dormant tumor cells and driving their re-entry into rapid proliferation cycle (44, 45).

### 2.2 Epigenetic modifications

The longevity and reversibility of tumor dormancy are precisely orchestrated by epigenetic modifications (19). Epigenetic modifications regulate chromatin structure and gene accessibility, influencing gene expression without altering the DNA sequence, and play an essential role in the induction, maintenance, and exit of tumor dormancy (46, 47). Among these, disruption in DNA methylation is characterized by increased methylation of tumor suppressor genes and decreased methylation of oncogenes. This may lead to the silencing of protective genes and the activation of harmful ones, thereby promoting tumor growth and recurrence (48). Similarly, alterations in histone acetylation and methylation levels can modify chromatin structure and gene activity, thereby influencing tumor cell behavior and evolutionary trajectories (49).

The receptor tyrosine kinase MERTK functions as a critical upstream signaling molecule regulating the equilibrium between histone methylation and dormancy. Cackowski et al. demonstrated in prostate cancer that knockdown of MERTK induced higher levels of histone H3K9me3 and H3K27me3, accompanied by upregulation of p27, NR2F1, SOX2, and NANOG, as well as G_1_/G_0_ arrest, all of which are associated with dormancy. The effects of MERTK on the cell cycle and histone methylation were reversed by the p38 inhibitor SB203580, indicating that MERTK regulates dormancy escape through a MAP kinase dependent mechanism involving p27 and histone methylation (50). The histone methyltransferases KMT2B and KMT2D play an essential role in maintaining dormancy phenotypes in breast cancer through the maintenance of H3K4 methylation homeostasis (51, 52). This mechanism exhibits cell-autonomous characteristics: breast cancer cells can maintain dormancy through H3K4 methyltransferases even after detachment from the bone marrow microenvironment. Functionally, H3K4 methyltransferases sustain the cell-autonomous regulatory program of cancer cells, enhancing their chemoresistance and maintaining cell cycle quiescence. Knockdown of KMT2B or KMT2D abrogates this braking mechanism, activating signaling pathways associated with proliferation, thereby driving DTCs out of dormancy and restoring chemosensitivity (52).

Histone acetylation modifications also play an essential role in dormancy regulation. Histone deacetylases (HDACs) condense chromatin structure, thereby reducing gene transcriptional accessibility. Conversely, HDAC inhibitors (HDACis) increase histone acetylation levels, shifting chromatin to an open and active state (53). In breast cancer, HDACis upregulate LIFR expression by increasing histone acetylation at the LIFR promoter, subsequently activating p38*β* and p21 signaling to establish a dormant phenotype. Notably, microenvironmental cues such as hypoxia or parathyroid hormone-related protein (PTHrP) suppress LIFR transcription by increasing H3K9me3 levels at the distal LIFR promoter, while HDACi treatment reverses this inhibitory effect and restores LIFR as well as its downstream dormancy-associated gene expression. Clinical studies indicate that HDACi induce the expression of pro-dormancy genes and correlate with prolonged survival in patients with breast cancer, suggesting that HDACi represents a potential therapeutic strategy for preventing recurrence through the promotion of dormancy (53).

### 2.3 Post-transcriptional regulation by non-coding RNAs

Non-coding RNAs (ncRNAs), predominantly long non-coding RNAs (lncRNAs) and microRNAs (miRNAs), constitute an intricate post-transcriptional regulatory network that profoundly influences dormant cell fate by modulating mRNA stability, translational efficiency, and alternative pre-mRNA splicing events (18, 54). LncRNAs contribute to tumor dormancy through versatile mechanisms encompassing cellular quiescence, metastasis, and CSC phenotypes. At the transcriptional level, NR2F1-AS1 has been demonstrated in estrogen receptor (ER)-positive breast cancer to drive tumor cells into a quiescence-like state by upregulating dormancy-inducing factors and pluripotency markers, thereby fostering recurrence (55). In terms of chromatin remodeling and stemness regulation, the lncRNA HOTAIR promotes CSC maintenance, metastatic dissemination, and chemoresistance through a tripartite mechanism involving PRC2/EZH2-mediated epigenetic silencing, the miR-34a/SOX2 stemness axis, and the p53/p21 cell cycle pathway. The high expression of HOTAIR in CSCs and its regulation of self-renewal capacity position it as a potential therapeutic target for overcoming tumor recurrence. Moreover, HOTAIR-mediated cell cycle regulation via the p53/p21 pathway suggests that it may play an important role in the dormancy-reactivation balance (56, 57). Collectively, these lncRNAs play critical roles in regulating tumor dormancy, survival, and metastasis, highlighting their potential as therapeutic targets.

MicroRNAs (miRNAs) also play essential roles in balancing pro- dormancy and anti-dormancy programs (58). Among pro-dormancy miRNAs, miR-190 induces quiescence by targeting angiogenesis-related genes, particularly in bone cancer and glioblastoma. Its upregulation substantially prolongs dormancy latency and suppresses the transition toward a proliferative phenotype (59). The miR-200 family (comprising miR-200a, miR-200b, miR-200c, miR-141, and miR-429) represents a critical regulator of breast cancer progression, and downregulation of this family is closely associated with epithelial-mesenchymal transition (EMT), whereas re-expression of the miR-200b/200a/429 cluster enforces a dormancy-like state *in vivo* (60, 61). Similarly, miR-335 suppresses the transcription factor SOX4, which not only impairs cancer cell migratory and invasive capacity but also triggers cell cycle arrest, establishing a permissive state for dormant colonization in distant organs (62). Furthermore, multiple miRNAs (miR-127, miR-197, and miR-223) participate in the maintenance of dormancy by targeting chemokines such as CXCL12 to suppress breast cancer cell proliferation (63).

Remarkably, specific miRNAs exert anti-dormancy or pro-proliferative functions by targeting essential cell cycle regulators. In leukemia, bone marrow niche cells (including stromal cells and osteoblasts) induce downregulation of miR-221/222 in acute lymphoblastic leukemia (ALL) cells, leading to upregulation of p27, a key negative regulator of cell cycle progression. Elevated p27 subsequently arrests tumor cells in G0 phase while conferring chemoresistance, thereby promoting tumor cell quiescence and residual survival. Conversely, forced overexpression of miR-221/222 reverses this effect by downregulating p27 (64). These miRNAs underscore the complex molecular landscape controlling tumor dormancy and illuminate their potential as therapeutic targets for preventing metastasis and recurrence.

### 2.4 Post-translational modification

Beyond transcriptional and post-transcriptional regulation, lactylation and ubiquitination have emerged as critical regulators of the induction, maintenance, and reversal of tumor dormancy. These modifications can rapidly and reversibly alter protein stability, activity, and interactions without changing the DNA sequence, enabling dormant cells to adapt to microenvironmental stress and therapeutic insults.

In triple-negative breast cancer (TNBC), glycolytic enzyme enolase 1 (ENO1) expression is positively correlated with elevated histone H4 lysine 12 lactylation (H4K12la), and the ENO1-lactate-H4K12la positive feedback loop drives the activation of quiescent breast cancer stem-like cells (65). In addition, the ubiquitin-proteasome system participates in dormancy regulation by controlling the stability of dormancy-associated proteins. For example, the F-box protein FBX8 directly binds to hypoxia-inducible factor 1*α* (HIF-1*α*), CDK4, and c-Myc through its Sec7 domain, mediating their ubiquitination and degradation, thereby inhibiting cell cycle progression, proliferation, and angiogenesis, and promoting dormancy in colorectal cancer liver metastases (66). In contrast, CSN8 increases HIF-1*α* mRNA expression by activating NF-*κ*B and stabilizes HIF-1*α* protein via de-ubiquitination, thereby inducing dormancy under hypoxia (67).

### 2.5 Metabolic and bioenergetic regulation

Conventional chemotherapy and radiotherapy predominantly target rapidly proliferating cells, whereas dormant cells can evade therapeutic killing due to their low proliferation and metabolic activity (68). Dormant tumor cells undergo profound metabolic reprogramming, characterized by reduced reliance on glycolysis and enhanced mitochondrial oxidative phosphorylation (OXPHOS) and fatty acid oxidation (FAO), thereby supporting ATP production and cellular homeostasis under hypoxia and conferring therapeutic tolerance (69). In CRC, FAO has been reported to promote ACLY expression and drive acetyl-CoA synthesis, which is transferred by p300 to accelerate H3K27 acetylation of the Nanog promoter. Subsequently, upregulated Nanog increases the transcription of p21 and p27, thereby promoting the entry of CRC cells into dormancy (70).

Autophagy is activated in dormant cells from a range of malignancies, including breast, ovarian, and pancreatic cancers, as well as osteosarcoma. Dormant cells display markedly enhanced autophagic activity, evidenced by a higher abundance of autophagosomes and autolysosomes, together with elevated expression of autophagy-related genes such as LC3, ATG4, ATG5, ATG7, and BECN1 (71). In head and neck squamous cell carcinoma, mitophagy triggers a dormant phenotype that shields cancer cells from cisplatin-induced cytotoxicity, and co-administration of autophagy inhibitors with cisplatin markedly improves treatment response (72). Furthermore, autophagic activation is tightly linked to suppression of the PI3 K/AKT/mTOR pathway; through the integrin/PI3 K/Akt/mTOR axis, redox signals stimulate autophagy and modulate transcription, thereby sustaining the dormant state (73, 74).

## 3 Microenvironmental regulation of dormancy and awakening

Tumor cell dormancy is an active biological process, rather than a passive state, orchestrated by both intracellular signals and microenvironmental cues (10, 22). The maintenance of dormancy and its awakening are dynamically regulated by multiple microenvironmental components, including hypoxia, the extracellular matrix, endothelial cells, fibroblasts, immune cells, and inflammatory signals. Disruption of this equilibrium (frequently triggered by chronic inflammation, stromal remodeling, or therapy-induced stress) can drive dormant cells to exit dormancy, re-enter the proliferation cycle, and give rise to metastatic lesions (75–77).

### 3.1 Hypoxia and the extracellular matrix

Hypoxia, a core microenvironmental cue, activates HIF-1*α*, which upregulates the expression of CKIs such as p21, thereby inducing G₀/G₁ cell cycle arrest (78). Under hypoxic conditions, dormant cells maintain homeostasis through metabolic reprogramming. In addition, remodeling of the extracellular matrix and altered ECM-mediated signaling are critical for dormancy regulation and contribute to recurrence and metastatic tumor formation (79). For example, fibronectin-mediated integrin *β*1 signaling triggers MLCK-dependent phosphorylation of myosin light chain, which in turn leads to cytoskeletal rearrangement, stress fiber formation, and the awakening of dormant cells (80). A fibrotic microenvironment induced by type I collagen deposition activates integrin *β*1 signaling, driving dormant cancer cells to exit dormancy and re-enter proliferation (20). Moreover, in a head and neck squamous carcinoma model, the microenvironment surrounding dormant cells is enriched with type III collagen, and exogenous supplementation of this collagen promotes both the entry into and maintenance of dormancy (81–83).

### 3.2 Endothelial cells, fibroblasts, and the perivascular niche

Dormant cancer cells reside in specialized “dormant niches,” where they are dynamically regulated by endothelial and stromal cells (27). In breast cancer models, quiescent microvascular endothelial cells secrete thrombospondin-1 (TSP-1), which induces cell-cycle arrest in cancer cells and thereby exerts a tumor-suppressive effect (84, 85). However, during angiogenesis, TSP-1 levels decline around nascent vessels, accompanied by the accumulation of factors such as TGF-*β*1 and periostin (POSTN), which disrupt the inhibitory microenvironment and awaken dormant cells (86–88).

In addition to endothelial cells, cancer-associated fibroblasts (CAFs) represent a key population in the tumor stroma that suppress immune cell function by cytokine and metabolite secretion, remodel the extracellular matrix, and establish physical barriers against drug and immune cell penetration, thereby compromising therapeutic efficacy (89–91). Notably, studies in breast cancer have demonstrated that CAFs can influence tumor cell metabolism by increasing oxidative phosphorylation, which facilitates exit from dormancy and promotes proliferation and metastasis (92). Collectively, these findings underscore that metabolic support from the stroma constitutes a key trigger for awakening (92).

### 3.3 Immune cells and immunological dormancy

Clinical and experimental evidence supports the involvement of the immune system in regulating cancer cell dormancy. For example, dormant DTCs have been observed in the bone marrow of breast cancer patients, accompanied by an increase in multiple immune subsets, including NK cells, macrophages, and T cells, suggesting that immune cells play an important role in dormancy regulation (93).

CD8+ T cells are crucial lymphocytes for tumor suppression. Depletion of CD8+ T cells is significantly associated with shortened dormancy duration in B cell lymphoma recurrence; studies have shown that CD8⁺ T cells promote the induction and maintenance of dormancy through the production of IFN-*γ* (94, 95). In addition, in a murine acute myeloid leukemia model, B7-H1 (PD-L1, the ligand for PD-1) or B7-1 (CD80, the ligand for CTLA-4) expression was shown to prolong tumor cell survival by suppressing CD8+ T cell-mediated killing, uncovering another mechanism by which CD8+ T cells contribute to cancer dormancy (96).

CD4⁺ T cells can exert anti-cancer effects by modulating the microenvironment and help CD8+ T cells enhance anti-tumor responses (97). In breast cancer patients and mouse lymphoma models, dormant DTCs coexist with increased CD4 + and CD8+ T cells (93, 98). CD4⁺ T cells (Th1) can arrest tumor growth and establish dormancy via IFN-*γ* and tumor necrosis factor p55 receptor (TNFR1) signaling (94, 99).

NK cells induce tumor cell death through cytotoxicity and cytokine production. In breast cancer liver metastasis, NK cells help maintain tumor cell dormancy through the IFN-*γ* signaling pathway, thereby preventing liver metastasis (100). Additionally, cytotoxicity mediated by perforin restrains the growth of cancer cells in host tissue, forcing them to stay dormant for at least 30 days (101).

Myeloid-derived suppressor cells (MDSCs) promote the reactivation of dormant DTCs during the establishment of pre-metastatic niches (102). MDSCs suppress T cell responses through direct cell–cell contact or the production of arginase, iNOS, ROS, and peroxynitrite, and promote metastasis by secreting MMP9, which increases VEGF bioavailability and disrupts angiogenic dormancy (103). In addition to MDSCs, macrophages modulate cancer cell dormancy through the TGF-*β*, WNT, and Hippo pathways (104).

### 3.4 Inflammatory signals

Chronic inflammation serves as a critical trigger of dormant cancer cell awakening. For instance, in the context of breast cancer liver metastasis, chronic liver inflammation activates hepatic stellate cells, promoting them to secrete the chemokine CXCL12, which in turn suppresses NK cells and promotes the reawakening and proliferation of dormant breast cancer cells (100). Additionally, NETs, formed by neutrophils in response to inflammatory stimuli, have been shown to reactivate dormant tumor cells (30). Indeed, surgical stimuli, among other triggers, can induce NET formation in breast cancer, thereby awakening dormant tumor cells and fostering metastatic outgrowth (Figure 1) (105, 106).

**Figure 1:**
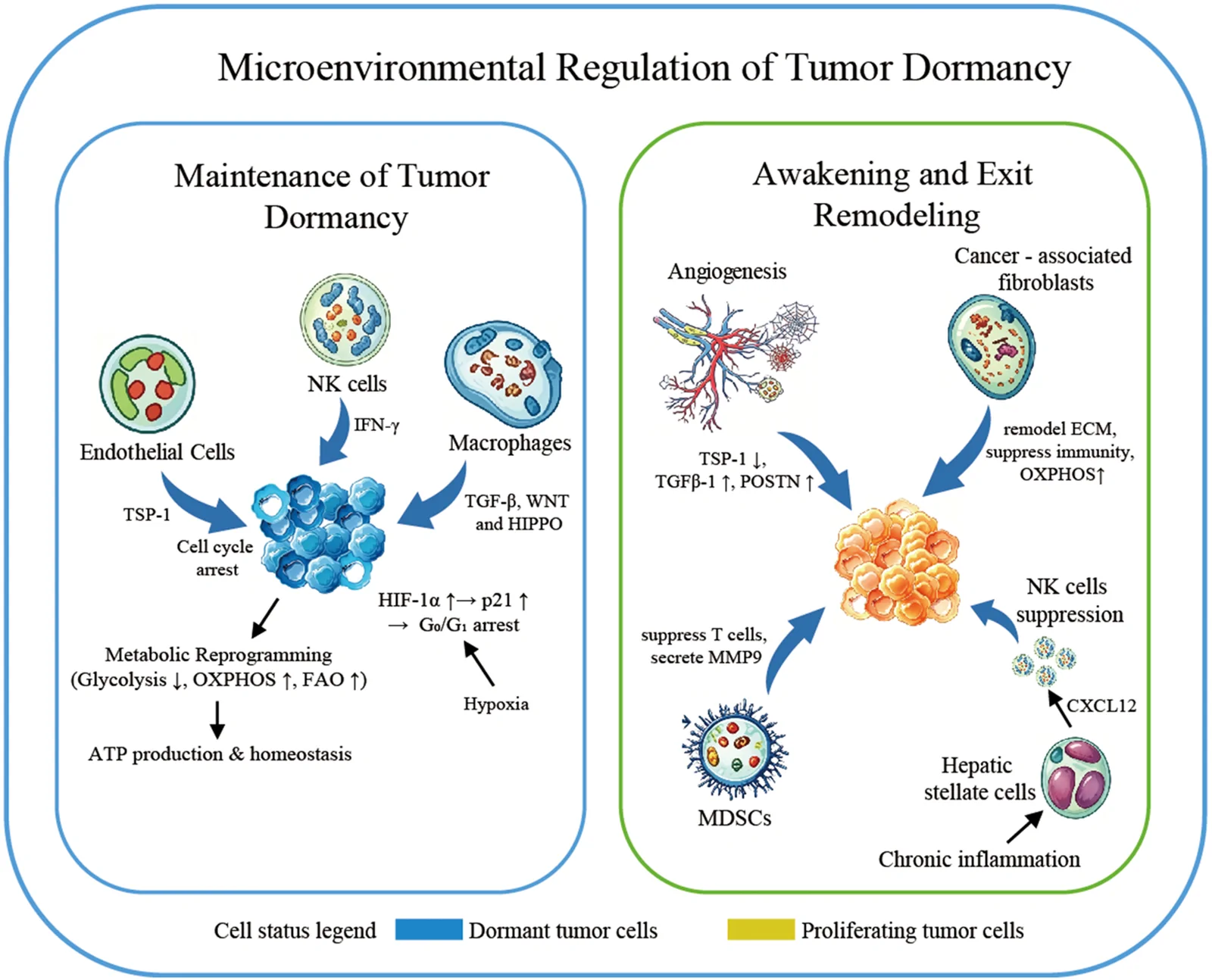
Regulation of tumor dormancy by the microenvironment. Tumor dormancy is regulated by bidirectional interactions between cancer cells and the microenvironment. Left panel (maintenance): Hypoxia activates HIF-1*α*, upregulates p21, and induces G₀/G₁ arrest. Endothelial cells secrete TSP-1 to induce cell cycle arrest. NK cells via IFN-*γ* maintain dormancy. Macrophages via TGF-*β*/WNT/Hippo pathways regulate dormancy. Right panel (awakening): Angiogenesis reduces TSP-1 and increases TGF-*β*1 and POSTN, disrupting the niche. CAFs remodel the ECM, suppress immunity, and enhance OXPHOS to promote awakening. MDSCs suppress T cells and secrete MMP9 to disrupt angiogenic dormancy. Chronic inflammation activates hepatic stellate cells to secrete CXCL12, which suppresses NK cells and promotes awakening. Blue = dormant tumor cells; orange = proliferating tumor cells.

### 3.5 Organ-specific heterogeneity of dormant niches

The maintenance and reactivation of dormant tumor cells are not governed by a uniform mechanism, but are profoundly shaped by the microenvironments of different organs (18). In the liver, dormant niches consist mainly of non-parenchymal cells (NPCs), including immune cells, stromal cells, endothelial cells, and hepatic stellate cells (107, 108). These cells modulate dormancy through direct physical contact and secreted factors. Upon liver injury, hepatic stellate cells produce large amounts of collagen, forming a fibrotic microenvironment; this extracellular matrix environment can induce metastatic outgrowth from dormant cells (20, 109, 110).

In the brain, endothelial cells and pericytes in the perivascular region have also been demonstrated to constitute the dormancy and metastatic niches in brain tissue for breast cancer cells (87, 111). A study on glioblastoma has shown that tumor cells in the bulk and at the margin exhibit distinct biological properties: marginal tumor cells are predominantly dormant glioblastoma cells and tend toward astrocyte-like differentiation, whereas dormant cells inside the bulk tumors resemble the features of injured neural progenitor-like cells, such as high expression of SRY-Box transcription factor 9 (SOX9) (112).

In summary, although the specific regulatory mechanisms of dormant niches in different organs have not been fully elucidated, these organ-specific differences indicate that directly extrapolating findings from one organ to another carries considerable risk; therapeutic strategies should therefore be tailored to specific organs and tumor types.

## 4 Therapeutic strategies targeting tumor dormancy

Given their pronounced therapeutic tolerance, dormant tumor cells are largely refractory to conventional treatments. Current research efforts have concentrated on three major strategies: long-term dormancy maintenance (Lock-and-Block), active awakening followed by sensitization and elimination (Awaken-and-Kill), and direct eradication (Direct Eradication) (113).

### 4.1 Lock-and-block strategy

Accumulating evidence indicates that residual tumor cells surviving therapy frequently acquire enhanced drug resistance. Consequently, an alternative paradigm has emerged, proposing that these cells enter into dormancy rather than targeted for immediate eradication (10, 114, 115). Building on this premise, this strategy aims to artificially reinforce pro-dormancy signals, thereby preventing dormant cancer cells from transitioning to a proliferative state (116). Specific approaches include kinase signal inhibition, such as suppressing uPAR, integrin *β*1, ERK, and Src kinase activities, which has been shown to induce DTCs to enter dormancy in preclinical models (117, 118). Preclinical studies have confirmed that either ERK or Src inhibitors effectively sustain breast cancer cell dormancy (20). However, clinical evidence for the efficacy of these inhibitors in sustaining dormancy remains limited. Nevertheless, this approach fails to eliminate dormant cancer cells, necessitating prolonged or even lifelong therapeutic regimens, which impose substantial economic burdens and raise toxicity concerns (75, 114).

### 4.2 Awaken-and-kill strategy

This approach aims to force dormant cells to re-enter the cell cycle, thereby overcoming their therapy resistant phenotype. For instance, preclinical studies have shown that neutralizing osteopontin (OPN) secreted by bone marrow osteoblasts using specific antibodies can disrupt the quiescence-promoting signal, thereby triggering the awakening of dormant leukemia cells (119). Similarly, administration of granulocyte colony-stimulating factor (G-CSF) can provoke dormant leukemia stem cells to re-enter the cell cycle, thereby significantly enhancing chemotherapy efficacy by disrupting the microenvironmental niches that maintain dormancy (120). However, this strategy entails severe safety concerns: if the combined chemotherapy fails to completely eliminate the reawakened cells, the residual DTCs may acquire enhanced aggressiveness (77). Consequently, in the absence of highly effective subsequent eradication, this strategy may paradoxically worsen patient prognosis (77, 121).

### 4.3 Direct eradication strategy

Developing agents that directly eliminate dormant cells represents a more intuitive therapeutic approach. For instance, autophagy inhibition has been shown to eliminate dormant cells. Vera-Ramirez et al. reported that pharmacological or genetic inhibition of autophagy (such as with chloroquine or ATG7 knockdown) substantially reduces the survival of dormant breast cancer cells and metastatic burden in mouse and human 3D *in vitro* and *in vivo* preclinical models of dormancy. Mechanistically, inhibition of autophagic flux in dormant breast cancer cells leads to the accumulation of damaged mitochondria and reactive oxygen species (ROS), resulting in apoptosis (122). However, once dormant cells re-enter a proliferative state, autophagy inhibition no longer affects their growth, underscoring that the therapeutic window for this intervention must be strictly confined to the dormant phase (69). Furthermore, ferroptosis induction has emerged as a novel strategy in preclinical studies (123). For instance, residual dormant breast cancer, lung adenocarcinoma, and ovarian cancer cells following drug treatment can be effectively eliminated *in vitro* and in mouse models by the glutathione peroxidase 4 (GPX4) inhibitors (RSL3 or ML210). Mechanistically, these dormant cells are highly dependent on GPX4 for survival. Thus, targeted GPX4 inhibition specifically triggers ferroptosis (Figure 2) (124, 125). It should be noted that these ferroptosis-inducing agents have not yet been tested in clinical trials for dormancy-targeting indications. However, this strategy remains constrained by current limitations in minimal residual disease detection and carries the risk of secondary resistance arising from incomplete elimination (68).

**Figure 2:**
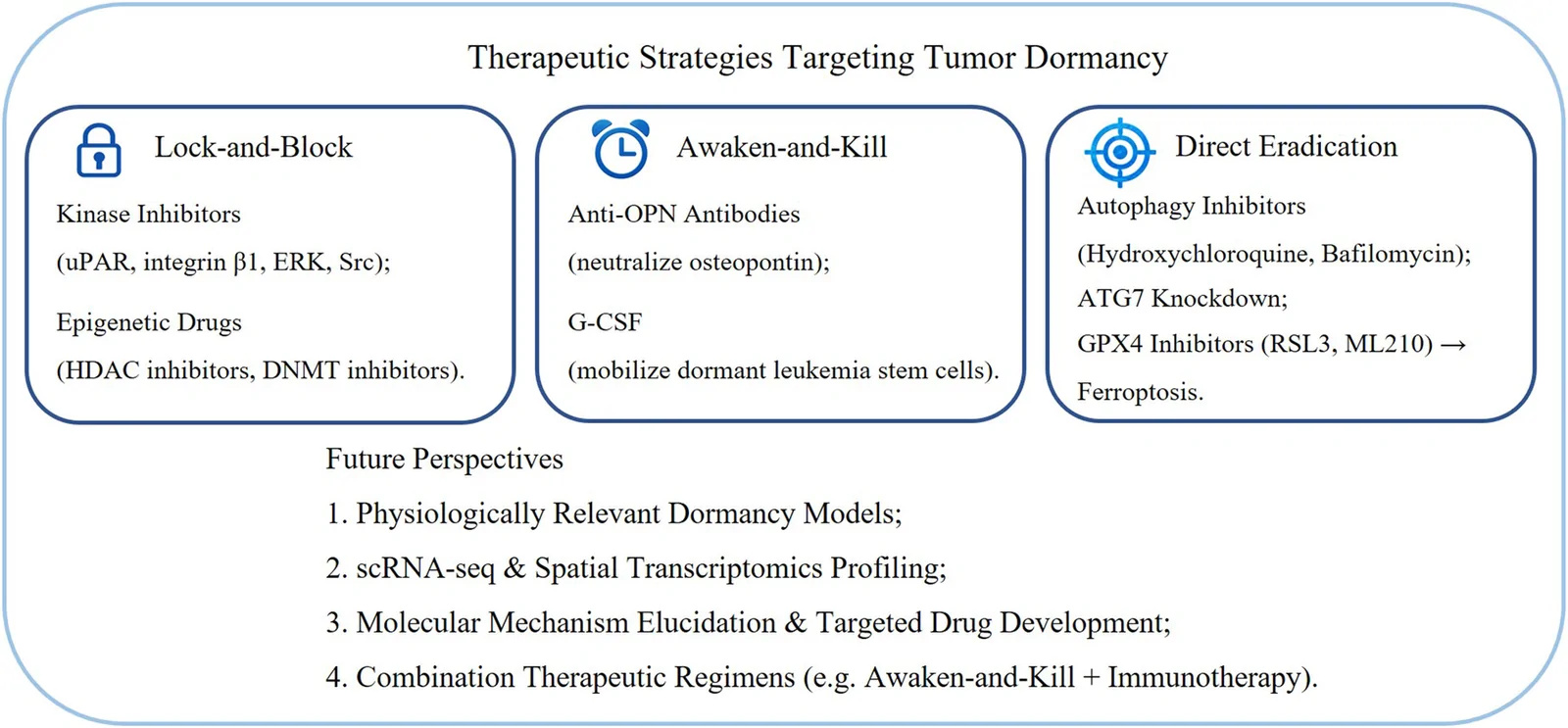
Therapeutic strategies targeting dormant tumor cells. Three strategies are outlined. Lock and Block: reinforces pro dormancy signals (kinase inhibitors targeting uPAR, integrin *β*1, ERK, Src, and epigenetic drugs) but cannot eliminate cells and requires long term use. Awaken and Kill: forces cell cycle re entry to restore therapy sensitivity (anti OPN antibodies, G CSF), but incomplete elimination may enhance aggressiveness. Direct Eradication: directly kills dormant cells (autophagy inhibitors, ATG7 knockdown, GPX4 inhibitors), but limited by MRD detection and secondary resistance. Future directions: physiologically relevant models, single cell/spatial transcriptomics, mechanistic studies, and combination therapies.

## 5 Conclusions and future perspectives

Dormant cancer cells serve as the principal reservoirs driving metastatic dissemination and long-term recurrence. Characterized by latent survival, intrinsic drug resistance, and reversible proliferation, they represent a major barrier to achieving cancer cure. An improved understanding of dormancy biology has not only elucidated their critical roles in mediating metastasis and recurrence, but also prompted the development of strategies such as “Awaken-and-Kill”, “Lock-and-Block”, and “Direct Eradication”, which provide novel approaches for countering tumor relapse.

However, clinical translation still remains hindered by several formidable challenges. First, owing to their sparse and disseminated distribution, dormant cancer cells evade conventional imaging modalities, rendering current diagnostic tools incapable of accurately assessing therapeutic responses. Second, dormant cancer cells exhibit profound heterogeneity across different tissues and cancer types, manifesting distinct molecular signatures and regulatory networks, which renders a universal treatment inherently implausible. Third, incomplete execution of the “Awaken-and-Kill” strategy may inadvertently provoke widespread artificial reactivation, potentially accelerating tumor progression and entailing substantial clinical safety risks.

To overcome these obstacles, future research should be directed toward the following directions. First, the development of more physiologically relevant dormancy models that faithfully recapitulate the entire process of dissemination, colonization, dormancy, and eventual awakening, thereby providing reliable platforms for mechanistic investigation and drug screening. Second, leveraging single-cell RNA sequencing (scRNA-seq) and spatial transcriptomics will be instrumental in deciphering the molecular architecture and inter-cellular heterogeneity of dormant cells. Third, continued elucidation of the core molecular mechanisms governing dormancy induction, maintenance, and exit, is imperative to facilitate the design of highly specific, low-toxicity targeted drugs. Fourth, rational exploration of combination regimens, such as integrating the “Awaken-and-Kill” strategy with immunotherapeutic interventions, holds promise for achieving synergistic effects, enhancing overall efficacy, and mitigating the risk of secondary resistance. Fifth, and most critically, most dormancy-targeting strategies discussed in this review remain at the preclinical stage. Although agents such as CDK4/6 inhibitors have been approved for other clinical indications, their specific application to eliminate or awaken dormant tumor cells has not been rigorously tested in dedicated clinical trials. The translation of ferroptosis inducers, autophagy modulators, and niche-targeting antibodies from bench to bedside faces substantial hurdles, including the lack of reliable biomarkers to identify dormant cells in patients and the absence of imaging modalities to monitor treatment response. Therefore, while the therapeutic strategies outlined here hold considerable promise, their clinical applicability remains largely hypothetical and requires validation in prospective clinical trials.

Ultimately, the clinical cure of tumors depends not only on the aggressive elimination of primary lesions and overt metastases but more critically, on the successful eradication of occult dormant seeds. As our understanding of the dormant molecular landscape and its associated microenvironmental regulatory circuits continue to deepen, safer and more effective comprehensive strategies are anticipated to emerge. Such approaches harbor the potential to fundamentally impede tumor metastasis and recurrence, thereby offering renewed hope for achieving durable long-term remission in cancer patients.

## References

[1] Zhang M Liu C Tu J Tang M Ashrafizadeh M Nabavi N. Advances in cancer immunotherapy: historical perspectives, current developments, and future directions. Mole Cancer. (2025) 24(1):136. 10.1186/s12943-025-02305-x PMC12057291 40336045

[2] Wang C Li J Chen J Wang Z Zhu G Song L. Multi-omics analyses reveal biological and clinical insights in recurrent stage I non-small cell lung cancer. Nat Commun. (2025) 16(1):1477. 10.1038/s41467-024-55068-2 39929832 PMC11811181

[3] Chen S Huang C Liao G Sun H Xie Y Liao C. Distinct single-cell immune ecosystems distinguish true and *de novo* HBV-related hepatocellular carcinoma recurrences. Gut. (2023) 72(6):1196–210. 10.1136/gutjnl-2022-328428 36596711

[4] Cañellas-Socias A Cortina C Hernando-Momblona X Palomo-Ponce S Mulholland EJ Turon G. Metastatic recurrence in colorectal cancer arises from residual EMP1(+) cells. Nature. (2022) 611(7936):603–13. 10.1038/s41586-022-05402-9 PMC7616986 36352230

[5] Shi ZD Sun Z Zhu ZB Liu X Chen JZ Hao L. Integrated single-cell and spatial transcriptomic profiling reveals higher intratumour heterogeneity and epithelial-fibroblast interactions in recurrent bladder cancer. Clin Transl Med. (2023) 13(7):e1338. 10.1002/ctm2.1338 37488671 PMC10366350

[6] Liang T Tao T Wu K Liu L Xu W Zhou D. Cancer-associated fibroblast-induced remodeling of tumor microenvironment in recurrent bladder cancer. Advanced science (weinheim, baden-wurttemberg. Germany. (2023) 10(31):e2303230. 10.1002/advs.202303230 PMC10625065 37743226

[7] Shen S Vagner S Robert C. Persistent cancer cells: the deadly survivors. Cell. (2020) 183(4):860–74. 10.1016/j.cell.2020.10.027 33186528

[8] Rozalén C Sangrador I Avalle S Blasco-Benito S Tzortzi P Sanz-Flores M. TIM3(+) breast cancer cells license immune evasion during micrometastasis outbreak. Cancer Cell. (2025) 43(8):1549–67. 10.1016/j.ccell.2025.06.015 PMC12416865 40645187

[9] Shaffer SM Dunagin MC Torborg SR Torre EA Emert B Krepler C. Rare cell variability and drug-induced reprogramming as a mode of cancer drug resistance. Nature. (2017) 546(7658):431–5. 10.1038/nature22794 28607484 PMC5542814

[10] Phan TG Croucher PI. The dormant cancer cell life cycle. Nat Rev Cancer. (2020) 20(7):398–411. 10.1038/s41568-020-0263-0 32488200

[11] Agudo J Aguirre-Ghiso JA Bhatia M Chodosh LA Correia AL Klein CA. Targeting cancer cell dormancy. Nat Rev Cancer. (2024) 24(2):97–104. 10.1038/s41568-023-00642-x 38062251 PMC11038906

[12] Hadfield G. The dormant cancer cell. Bri Med J. (1954) 2(4888):607–10. 10.1136/bmj.2.4888.607 PMC2078920 13190204

[13] Ganesh K Massagué J. Targeting metastatic cancer. Nat Med. (2021) 27(1):34–44. 10.1038/s41591-020-01195-4 33442008 PMC7895475

[14] Massagué J Ganesh K. Metastasis-initiating cells and ecosystems. Cancer Disc. (2021) 11(4):971–94. 10.1158/2159-8290.Cd-21-0010 PMC8030695 33811127

[15] Dalla E Sreekumar A Aguirre-Ghiso JA Chodosh LA. Dormancy in breast cancer. Cold Spring Harbor Perspect Med. (2023) 13(11):a041331. 10.1101/cshperspect.a041331 PMC10562521 37037598

[16] Gerstberger S Jiang Q Ganesh K. Metastasis. Cell. (2023) 186(8):1564–79. 10.1016/j.cell.2023.03.003 37059065 PMC10511214

[17] Ren X Song M Liu X He W. Circulating tumor cells: mechanisms and clinical significance in colorectal cancer metastasis. Mole Cancer. (2025) 24(1):242. 10.1186/s12943-025-02465-w PMC12495746 41044624

[18] Liu R Zhao Y Su S Kwabil A Njoku PC Yu H. Unveiling cancer dormancy: intrinsic mechanisms and extrinsic forces. Cancer Lett. (2024) 591:216899. 10.1016/j.canlet.2024.216899 38649107 PMC13001072

[19] Tufail M Jiang CH Li N. Tumor dormancy and relapse: understanding the molecular mechanisms of cancer recurrence. Mil Med Res. (2025) 12(1):7. 10.1186/s40779-025-00595-2 39934876 PMC11812268

[20] Barkan D El Touny LH Michalowski AM Smith JA Chu I Davis AS. Metastatic growth from dormant cells induced by a col-I-enriched fibrotic environment. Cancer Res. (2010) 70(14):5706–16. 10.1158/0008-5472.Can-09-2356 20570886 PMC3436125

[21] Endo H Inoue M. Dormancy in cancer. Cancer Sci. (2019) 110(2):474–80. 10.1111/cas.13917 30575231 PMC6361606

[22] Aguirre-Ghiso JA. Models, mechanisms and clinical evidence for cancer dormancy. Nat Rev Cancer. (2007) 7(11):834–46. 10.1038/nrc2256 17957189 PMC2519109

[23] Bergers G Benjamin LE. Tumorigenesis and the angiogenic switch. Nat Rev Cancer. (2003) 3(6):401–10. 10.1038/nrc1093 12778130

[24] Indraccolo S Stievano L Minuzzo S Tosello V Esposito G Piovan E. Interruption of tumor dormancy by a transient angiogenic burst within the tumor microenvironment. Proc Natl Acad Sci U S A. (2006) 103(11):4216–21. 10.1073/pnas.0506200103 16537511 PMC1449673

[25] Yeh AC Ramaswamy S. Mechanisms of cancer cell dormancy–another hallmark of cancer? Cancer Res. (2015) 75(23):5014–22. 10.1158/0008-5472.Can-15-1370 26354021 PMC4668214

[26] Swann JB Smyth MJ. Immune surveillance of tumors. J Clin Invest. (2007) 117(5):1137–46. 10.1172/jci31405 17476343 PMC1857231

[27] Ghajar CM. Metastasis prevention by targeting the dormant niche. Nat Rev Cancer. (2015) 15(4):238–47. 10.1038/nrc3910 25801619 PMC4842412

[28] Koebel CM Vermi W Swann JB Zerafa N Rodig SJ Old LJ. Adaptive immunity maintains occult cancer in an equilibrium state. Nature. (2007) 450(7171):903–7. 10.1038/nature06309 18026089

[29] Basu S Dong Y Kumar R Jeter C Tang DG. Slow-cycling (dormant) cancer cells in therapy resistance, cancer relapse and metastasis. Sem Cancer Biol. (2022) 78:90–103. 10.1016/j.semcancer.2021.04.021 PMC8576068 33979674

[30] Albrengues J Shields MA Ng D Park CG Ambrico A Poindexter ME. Neutrophil extracellular traps produced during inflammation awaken dormant cancer cells in mice. Science (New York, NY). (2018) 361(6409):eaao4227. 10.1126/science.aao4227 PMC6777850 30262472

[31] He J Zhou Z Cheng G Li H Tu J Fan Z. Neutrophil extracellular traps in the tumor microenvironment, metastasis, therapy, and beyond: advances, challenges, and perspectives. J Hematol Oncol. (2026) 19(1):29. 10.1186/s13045-026-01789-4 41987275 PMC13130571

[32] He D Wu Q Tian P Liu Y Jia Z Li Z. Chemotherapy awakens dormant cancer cells in lung by inducing neutrophil extracellular traps. Cancer Cell. (2025) 43(9):1622–36. 10.1016/j.ccell.2025.06.007 40614736

[33] Zhang J Zhang J Han L Wu S Li J Eaton EN. Inflammation awakens dormant cancer cells by modulating the epithelial-mesenchymal phenotypic state. Proc Natl Acad Sci U S A. (2025) 122(36):e2515009122. 10.1073/pnas.2515009122 40901881 PMC12435312

[34] Goddard ET Linde MH Srivastava S Klug G Shabaneh TB Iannone S. Immune evasion of dormant disseminated tumor cells is due to their scarcity and can be overcome by T cell immunotherapies. Cancer Cell. (2024) 42(1):119–134.e12. 10.1016/j.ccell.2023.12.011 38194912 PMC10864018

[35] Adam-Artigues A Valencia Salazar LE Aguirre-Ghiso JA. Immune evasion by dormant disseminated cancer cells: a Fermi paradox? Cancer Cell. (2024) 42(1):13–5. 10.1016/j.ccell.2023.12.017 38194913 PMC11034720

[36] Overton KW Spencer SL Noderer WL Meyer T Wang CL. Basal p21 controls population heterogeneity in cycling and quiescent cell cycle states. Proc Natl Acad Sci U S A. (2014) 111(41):E4386–93. 10.1073/pnas.1409797111 25267623 PMC4205626

[37] Gonzalez L Domingo-Muelas A Duart-Abadia P Nuñez M Mikolcevic P Llonch E. The atypical CDK activator RingoA/Spy1 regulates exit from quiescence in neural stem cells. iScience. (2023) 26(3):106202. 10.1016/j.isci.2023.106202 36876138 PMC9982312

[38] Weston WA Barr AR. A cell cycle centric view of tumour dormancy. Bri J Cancer. (2023) 129(10):1535–45. 10.1038/s41416-023-02401-z PMC10645753 37608096

[39] Litovchick L Florens LA Swanson SK Washburn MP DeCaprio JA. DYRK1A protein kinase promotes quiescence and senescence through DREAM complex assembly. Genes Dev. (2011) 25(8):801–13. 10.1101/gad.2034211 21498570 PMC3078706

[40] Khalil BD Sanchez R Rahman T Rodriguez-Tirado C Moritsch S Martinez AR. An NR2F1-specific agonist suppresses metastasis by inducing cancer cell dormancy. J Exp Med. (2022) 219(1):e20210836. 10.1084/jem.20210836 34812843 PMC8614154

[41] Sosa MS Parikh F Maia AG Estrada Y Bosch A Bragado P. NR2F1 controls tumour cell dormancy via SOX9- and RARβ-driven quiescence programmes. Nat Commun. (2015) 6:6170. 10.1038/ncomms7170 25636082 PMC4313575

[42] Liu Y Zhang P Wu Q Fang H Wang Y Xiao Y. Long non-coding RNA NR2F1-AS1 induces breast cancer lung metastatic dormancy by regulating NR2F1 and ΔNp63. Nat Commun. (2021) 12(1):5232. 10.1038/s41467-021-25552-0 34475402 PMC8413371

[43] Aguirre-Ghiso JA Ossowski L Rosenbaum SK. Green fluorescent protein tagging of extracellular signal-regulated kinase and p38 pathways reveals novel dynamics of pathway activation during primary and metastatic growth. Cancer Res. (2004) 64(20):7336–45. 10.1158/0008-5472.Can-04-0113 15492254

[44] Bragado P Estrada Y Parikh F Krause S Capobianco C Farina HG. TGF-β2 dictates disseminated tumour cell fate in target organs through TGF-β-RIII and p38α/β signalling. Nat Cell Biol. (2013) 15(11):1351–61. 10.1038/ncb2861 24161934 PMC4006312

[45] Wang Z Elbanna Y Godet I. TGF-β induces an atypical EMT to evade immune mechanosurveillance in lung adenocarcinoma dormant metastasis. Nat Cancer. (2026) 7(1):131–49. 10.1038/s43018-025-01094-y 41492090 PMC12858408

[46] Ferrer AI Trinidad JR Sandiford O Etchegaray JP Rameshwar P. Epigenetic dynamics in cancer stem cell dormancy. Cancer Metastasis Rev. (2020) 39(3):721–38. 10.1007/s10555-020-09882-x 32394305

[47] Robinson NJ Parker KA Schiemann WP. Epigenetic plasticity in metastatic dormancy: mechanisms and therapeutic implications. Ann Transl Med. (2020) 8(14):903. 10.21037/atm.2020.02.177 32793747 PMC7396775

[48] Henrique R Costa VL Cerveira N Carvalho AL Hoque MO Ribeiro FR. Hypermethylation of cyclin D2 is associated with loss of mRNA expression and tumor development in prostate cancer. J Mole Med (Berlin, Germany). (2006) 84(11):911–8. 10.1007/s00109-006-0099-4 17016690

[49] Li Z Zhao B Qin C Wang Y Li T Wang W. Chromatin dynamics in digestive system cancer: commander and regulator. Front Oncol. (2022) 12:935877. 10.3389/fonc.2022.935877 35965507 PMC9372441

[50] Cackowski FC Eber MR Rhee J Decker AM Yumoto K Berry JE. Mer tyrosine kinase regulates disseminated prostate cancer cellular dormancy. J Cell Biochem. (2017) 118(4):891–902. 10.1002/jcb.25768 27753136 PMC5296338

[51] Sandiford OA Donnelly RJ El-Far MH Burgmeyer LM Sinha G Pamarthi SH. Mesenchymal stem cell-secreted extracellular vesicles instruct stepwise dedifferentiation of breast cancer cells into dormancy at the bone marrow perivascular region. Cancer Res. (2021) 81(6):1567–82. 10.1158/0008-5472.Can-20-2434 33500249

[52] Ferrer-Diaz AI Sinha G Petryna A Gonzalez-Bermejo R Kenfack Y Adetayo O. Revealing role of epigenetic modifiers and DNA oxidation in cell-autonomous regulation of cancer stem cells. Cell Commun Sign. (2024) 22(1):119. 10.1186/s12964-024-01512-1 PMC10863086 38347590

[53] Zeng H Qu J Jin N Xu J Lin C Chen Y. Feedback activation of leukemia inhibitory factor receptor limits response to histone deacetylase inhibitors in breast cancer. Cancer Cell. (2016) 30(3):459–73. 10.1016/j.ccell.2016.08.001 27622335

[54] Parker KA Robinson NJ Schiemann WP. The role of RNA processing and regulation in metastatic dormancy. Semin Cancer Biol. (2022) 78:23–34. 10.1016/j.semcancer.2021.03.020 33775829 PMC8464634

[55] Calle A S Yamamoto T Kawamura Y Hironaka-Mitsuhashi A Ono M Tsuda H. Long non-coding NR2F1-AS1 is associated with tumor recurrence in estrogen receptor-positive breast cancers. Mole Oncol. (2020) 14(9):2271–87. 10.1002/1878-0261.12704 PMC7463365 32392629

[56] Wang J Liu X Li P Wang J Shu Y Zhong X. Long noncoding RNA HOTAIR regulates the stemness of breast cancer cells via activation of the NF-κB signaling pathway. J Biol Chem. (2022) 298(12):102630. 10.1016/j.jbc.2022.102630 36273585 PMC9691943

[57] Kogo R Shimamura T Mimori K Kawahara K Imoto S Sudo T. Long noncoding RNA HOTAIR regulates polycomb-dependent chromatin modification and is associated with poor prognosis in colorectal cancers. Cancer Res. (2011) 71(20):6320–6. 10.1158/0008-5472.Can-11-1021 21862635

[58] Hussain MS Moglad E Afzal M Gupta G Hassan Almalki W Kazmi I. Non-coding RNA mediated regulation of PI3 K/akt pathway in hepatocellular carcinoma: therapeutic perspectives. Pathol Res Pract. (2024) 258:155303. 10.1016/j.prp.2024.155303 38728793

[59] Almog N Briggs C Beheshti A Ma L Wilkie KP Rietman E. Transcriptional changes induced by the tumor dormancy-associated microRNA-190. Transcription. (2013) 4(4):177–91. 10.4161/trns.25558 23863200 PMC3977918

[60] Watson KL Jones RA Bruce A Moorehead RA. The miR-200b/200a/429 cluster prevents metastasis and induces dormancy in a murine claudin-low mammary tumor cell line. Exp Cell Res. (2018) 369(1):17–26. 10.1016/j.yexcr.2018.04.024 29702103

[61] Tiram G Segal E Krivitsky A Shreberk-Hassidim R Ferber S Ofek P. Identification of dormancy-associated MicroRNAs for the design of osteosarcoma-targeted dendritic polyglycerol nanopolyplexes. ACS Nano. (2016) 10(2):2028–45. 10.1021/acsnano.5b06189 26815014

[62] Tavazoie SF Alarcón C Oskarsson T Padua D Wang Q Bos PD. Endogenous human microRNAs that suppress breast cancer metastasis. Nature. (2008) 451(7175):147–52. 10.1038/nature06487 18185580 PMC2782491

[63] Lim PK Bliss SA Patel SA Taborga M Dave MA Gregory LA. Gap junction-mediated import of microRNA from bone marrow stromal cells can elicit cell cycle quiescence in breast cancer cells. Cancer Res. (2011) 71(5):1550–60. 10.1158/0008-5472.Can-10-2372 21343399

[64] Moses BS Evans R Slone WL Piktel D Martinez I Craig MD. Bone marrow microenvironment niche regulates miR-221/222 in acute lymphoblastic leukemia. Mole Cancer Res. (2016) 14(10):909–19. 10.1158/1541-7786.Mcr-15-0474 PMC5065748 27358112

[65] Liu S Ma Y Zeng Y Lv D Hou Z Huang J. Glycolytic enolase 1-induced H4K12 lactylation activates quiescent breast cancer stem-like cells in triple-negative breast cancer. Signal Transduct Target Ther. (2026) 11(1):341. 10.1038/s41392-026-02916-w 42624838 PMC13493833

[66] Zhu X Wang F Wu X Li Z Wang Z Ren X. FBX8 promotes metastatic dormancy of colorectal cancer in liver. Cell Death Dis. (2020) 11(8):622. 10.1038/s41419-020-02870-7 32796813 PMC7427987

[67] Ju S Wang F Wang Y Ju S. CSN8 is a key regulator in hypoxia-induced epithelial-mesenchymal transition and dormancy of colorectal cancer cells. Molec Cancer. (2020) 19(1):168. 10.1186/s12943-020-01285-4 PMC7708218 33261601

[68] Hao S Guo Y Huang Q Gan L Chen C Li Q. Targeting dormant cancer cells: ferroptosis as a precision therapeutic strategy. Cell Mole Biol Lett. (2026) 31(1):68. 10.1186/s11658-026-00895-y PMC13126796 41862812

[69] Wang Y Liu L Zhang X Liang T Bai X. Cancer dormancy and metabolism: from molecular insights to translational opportunities. Cancer Lett. (2025) 635:218097. 10.1016/j.canlet.2025.218097 41135858

[70] Zhang M Peng R Wang H Yang Z Zhang H Zhang Y. Nanog mediated by FAO/ACLY signaling induces cellular dormancy in colorectal cancer cells. Cell Death Dis. (2022) 13(2):159. 10.1038/s41419-022-04606-1 35177584 PMC8854412

[71] Ferdousmakan S Mansourian D Seyedi Asl FS Fathi Z Maleki-Sheikhabadi F Afjadi MN. Autophagy induced by metabolic processes leads to solid tumor cell metastatic dormancy and recurrence. Med Oncol (Northwood, London, England). (2025) 42(3):62. 10.1007/s12032-025-02607-6 39899220

[72] Sun Y Chen Y Liu Z Wang J Bai J Du R. Mitophagy-mediated tumor dormancy protects cancer cells from chemotherapy. Biomedicines. (2024) 12(2):305. 10.3390/biomedicines12020305 38397907 PMC10886527

[73] Wang H Wang N Xu D Ma Q Chen Y Xu S. Oxidation of multiple MiT/TFE transcription factors links oxidative stress to transcriptional control of autophagy and lysosome biogenesis. Autophagy. (2020) 16(9):1683–96. 10.1080/15548627.2019.1704104 31826695 PMC8386635

[74] Qiu Y Qiu S Deng L Nie L Gong L Liao X. Biomaterial 3D collagen I gel culture model: a novel approach to investigate tumorigenesis and dormancy of bladder cancer cells induced by tumor microenvironment. Biomaterials. (2020) 256:120217. 10.1016/j.biomaterials.2020.120217 32736172

[75] Manjili MH Khazaie K. Pattern recognition of tumor dormancy and relapse beyond cell-intrinsic and cell-extrinsic pathways. Sem Cancer Biol. (2022) 78:1–4. 10.1016/j.semcancer.2021.12.008 PMC12964419 34990835

[76] Drapela S Garcia BM Gomes AP Correia AL. Metabolic landscape of disseminated cancer dormancy. Trends Cancer. (2025) 11(4):321–33. 10.1016/j.trecan.2024.10.005 39510896 PMC11981868

[77] Jiao Y Yu Y Zheng M Yan M Wang J Zhang Y. Dormant cancer cells and polyploid giant cancer cells: the roots of cancer recurrence and metastasis. Clin Transl Med. (2024) 14(2):e1567. 10.1002/ctm2.1567 38362620 PMC10870057

[78] Gardner LB Li Q Park MS Flanagan WM Semenza GL Dang CV. Hypoxia inhibits G1/S transition through regulation of p27 expression. J Biol Chem. (2001) 276(11):7919–26. 10.1074/jbc.M010189200 11112789

[79] Winkler J Abisoye-Ogunniyan A Metcalf KJ Werb Z. Concepts of extracellular matrix remodelling in tumour progression and metastasis. Nat Commun. (2020) 11(1):5120. 10.1038/s41467-020-18794-x 33037194 PMC7547708

[80] Barkan D Kleinman H Simmons JL Asmussen H Kamaraju AK Hoenorhoff MJ. Inhibition of metastatic outgrowth from single dormant tumor cells by targeting the cytoskeleton. Cancer Res. (2008) 68(15):6241–50. 10.1158/0008-5472.Can-07-6849 18676848 PMC2561279

[81] Di Martino JS Nobre AR Mondal C Taha I Farias EF Fertig EJ. A tumor-derived type III collagen-rich ECM niche regulates tumor cell dormancy. Nat Cancer. (2022) 3(1):90–107. 10.1038/s43018-021-00291-9 35121989 PMC8818089

[82] Leitinger B. Pulling the strings of tumor collagen. Nat Cancer. (2022) 3(1):9–10. 10.1038/s43018-021-00323-4 35121996

[83] Sun X Wu B Chiang HC Deng H Zhang X Xiong W. Tumour DDR1 promotes collagen fibre alignment to instigate immune exclusion. Nature. (2021) 599(7886):673–8. 10.1038/s41586-021-04057-2 34732895 PMC8839149

[84] Petrik J Lauks S Garlisi B Lawler J. Thrombospondins in the tumor microenvironment. Sem Cell Dev Biol. (2024) 155(Pt B):3–11. 10.1016/j.semcdb.2023.05.010 37286406

[85] Longhi E Carminati L Carlessi E Belotti D Taraboletti G. Thrombospondin-1 in drug activity and tumor response to therapies. Sem Cell Dev Biol. (2024) 155(Pt B):45–51. 10.1016/j.semcdb.2023.06.009 37414720

[86] Lopes-Bastos B Jin L Ruge F Owen S Sanders A Cogle C. Association of breast carcinoma growth with a non-canonical axis of IFN*γ*/IDO1/TSP1. Oncotarget. (2017) 8(49):85024–39. 10.18632/oncotarget.18781 29156701 PMC5689591

[87] Ghajar CM Peinado H Mori H Matei IR Evason KJ Brazier H. The perivascular niche regulates breast tumour dormancy. Nat Cell Biol. (2013) 15(7):807–17. 10.1038/ncb2767 23728425 PMC3826912

[88] Oria VO Erler JT. Tumor angiocrine signaling: novel targeting opportunity in cancer. Cells. (2023) 12(20):2510. 10.3390/cells12202510 37887354 PMC10605017

[89] Li J Liu H Guo Q Zhang Y Li J Diao T. Single-cell screens identify ADAM12 as a fibroblast checkpoint impeding anti-tumor immunity. Cancer Cell. (2026) 44(2):424–42. 10.1016/j.ccell.2025.12.018 41544628

[90] Wu Y Li S Yu H Zhang S Yan L Guan X. Integrative single-cell and spatial transcriptomics analysis reveals ECM-remodeling cancer-associated fibroblast-derived POSTN as a key mediator in pancreatic ductal adenocarcinoma progression. Int J Biol Sci. (2025) 21(8):3573–96. 10.7150/ijbs.108618 40520021 PMC12160862

[91] Sahai E Astsaturov I Cukierman E DeNardo DG Egeblad M Evans RM. A framework for advancing our understanding of cancer-associated fibroblasts. Nat Rev Cancer. (2020) 20(3):174–86. 10.1038/s41568-019-0238-1 31980749 PMC7046529

[92] Sansone P Savini C Kurelac I Chang Q Amato LB Strillacci A. Packaging and transfer of mitochondrial DNA via exosomes regulate escape from dormancy in hormonal therapy-resistant breast cancer. Proc Natl Acad Sci U S A. (2017) 114(43):E9066–75. 10.1073/pnas.1704862114 29073103 PMC5664494

[93] Feuerer M Rocha M Bai L Umansky V Solomayer EF Bastert G. Enrichment of memory T cells and other profound immunological changes in the bone marrow from untreated breast cancer patients. Int J Cancer. (2001) 92(1):96–105. 10.1002/1097-0215 11279612

[94] Wang HF Wang SS Huang MC Liang XH Tang YJ Tang YL. Targeting immune-mediated dormancy: a promising treatment of cancer. Front Oncol. (2019) 9:498. 10.3389/fonc.2019.00498 31297335 PMC6607988

[95] Corthay A Bakacs T Thangavelu G Anderson CC. Tackling cancer cell dormancy: insights from immune models, and transplantation. Sem Cancer Biol. (2022) 78:5–16. 10.1016/j.semcancer.2021.02.002 33582171

[96] Saudemont A Quesnel B. In a model of tumor dormancy, long-term persistent leukemic cells have increased B7-H1 and B7.1 expression and resist CTL-mediated lysis. Blood. (2004) 104(7):2124–33. 10.1182/blood-2004-01-0064 15191948

[97] Borst J Ahrends T Bąbała N Melief CJM Kastenmüller W. CD4(+) T cell help in cancer immunology and immunotherapy. Nat Rev Immunol. (2018) 18(10):635–47. 10.1038/s41577-018-0044-0 30057419

[98] Mahnke YD Schwendemann J Beckhove P Schirrmacher V. Maintenance of long-term tumour-specific T-cell memory by residual dormant tumour cells. Immunology. (2005) 115(3):325–36. 10.1111/j.1365-2567.2005.02163.x 15946250 PMC1782166

[99] Müller-Hermelink N Braumüller H Pichler B Wieder T Mailhammer R Schaak K. TNFR1 signaling and IFN-gamma signaling determine whether T cells induce tumor dormancy or promote multistage carcinogenesis. Cancer Cell. (2008) 13(6):507–18. 10.1016/j.ccr.2008.04.001 18538734

[100] Correia AL Guimaraes JC Auf der Maur P De Silva D Trefny MP Okamoto R. Hepatic stellate cells suppress NK cell-sustained breast cancer dormancy. Nature. (2021) 594(7864):566–71. 10.1038/s41586-021-03614-z 34079127

[101] Brodbeck T Nehmann N Bethge A Wedemann G Schumacher U. Perforin-dependent direct cytotoxicity in natural killer cells induces considerable knockdown of spontaneous lung metastases and computer modelling-proven tumor cell dormancy in a HT29 human colon cancer xenograft mouse model. Mole Cancer. (2014) 13:244. 10.1186/1476-4598-13-244 PMC4239380 25373310

[102] Kitamura T Qian BZ Pollard JW. Immune cell promotion of metastasis. Nat Rev Immunol. (2015) 15(2):73–86. 10.1038/nri3789 25614318 PMC4470277

[103] Qu P Wang LZ Lin PC. Expansion and functions of myeloid-derived suppressor cells in the tumor microenvironment. Cancer Lett. (2016) 380(1):253–6. 10.1016/j.canlet.2015.10.022 26519756 PMC7477794

[104] Zhang H Ding Y Gu L Guo A Duan W Wang X. Macrophage regulation of tumor cell dormancy and the dormant niche: an overview in solid tumors. Cancer Metast Rev. (2025) 44(3):63. 10.1007/s10555-025-10280-4 40762813

[105] Tohme S Yazdani HO Al-Khafaji AB Chidi AP Loughran P Mowen K. Neutrophil extracellular traps promote the development and progression of liver metastases after surgical stress. Cancer Res. (2016) 76(6):1367–80. 10.1158/0008-5472.Can-15-1591 26759232 PMC4794393

[106] Park J Wysocki RW Amoozgar Z Maiorino L Fein MR Jorns J. Cancer cells induce metastasis-supporting neutrophil extracellular DNA traps. Sci Transl Med. (2016) 8(361):361ra138. 10.1126/scitranslmed.aag1711 27798263 PMC5550900

[107] Wheeler SE Clark AM Taylor DP Young CL Pillai VC Stolz DB. Spontaneous dormancy of metastatic breast cancer cells in an all human liver microphysiologic system. Bri J Cancer. (2014) 111(12):2342–50. 10.1038/bjc.2014.533 PMC4264444 25314052

[108] Clark AM Wheeler SE Young CL Stockdale L Shepard Neiman J Zhao W. A liver microphysiological system of tumor cell dormancy and inflammatory responsiveness is affected by scaffold properties. Lab Chip. (2016) 17(1):156–68. 10.1039/c6lc01171c 27910972 PMC5242229

[109] Barkan D Green JE Chambers AF. Extracellular matrix: a gatekeeper in the transition from dormancy to metastatic growth. Eur J Cancer (Oxford, England: 1990). (2010) 46(7):1181–8. 10.1016/j.ejca.2010.02.027 PMC2856784 20304630

[110] Di Martino JS Akhter T Bravo-Cordero JJ. Remodeling the ECM: implications for metastasis and tumor dormancy. Cancers (Basel). (2021) 13(19):4916. 10.3390/cancers13194916 34638400 PMC8507703

[111] Jiang Z Zhou J Li L Liao S He J Zhou S. Pericytes in the tumor microenvironment. Cancer Lett. (2023) 556:216074. 10.1016/j.canlet.2023.216074 36682706

[112] Garcia-Diaz C Pöysti A Mereu E Clements MP Brooks LJ Galvez-Cancino F. Glioblastoma cell fate is differentially regulated by the microenvironments of the tumor bulk and infiltrative margin. Cell Rep. (2023) 42(5):112472. 10.1016/j.celrep.2023.112472 37149862

[113] Recasens A Munoz L. Targeting cancer cell dormancy. Trends Pharmacol Sci. (2019) 40(2):128–41. 10.1016/j.tips.2018.12.004 30612715

[114] Cackowski FC Heath EI. Prostate cancer dormancy and recurrence. Cancer Lett. (2022) 524:103–8. 10.1016/j.canlet.2021.09.037 34624433 PMC8694498

[115] Janowska A Iannone M Fidanzi C Romanelli M Filippi L Del Re M. The genetic basis of dormancy and awakening in cutaneous metastatic melanoma. Cancers (Basel). (2022) 14(9):2104. 10.3390/cancers14092104 35565234 PMC9102235

[116] Demicheli R Desmedt C Piccart M Biganzoli E. Tumor dormancy at bedside: a late awakening. Breast (Edinburgh, Scotland). (2019) 45:61–3. 10.1016/j.breast.2019.03.001 30878882

[117] Barkan D Chambers AF. *β*1-integrin: a potential therapeutic target in the battle against cancer recurrence. Clin Cancer Res. (2011) 17(23):7219–23. 10.1158/1078-0432.Ccr-11-0642 21900388

[118] Lin H Hassan Safdar M Washburn S Saeed SA Dickerhoff J Ayers M. Fibroblast growth receptor 1 is regulated by G-quadruplex in metastatic breast cancer. Commun Biol. (2024) 7(1):963. 10.1038/s42003-024-06602-x 39122837 PMC11316068

[119] Boyerinas B Zafrir M Yesilkanal AE Price TT Hyjek EM Sipkins DA. Adhesion to osteopontin in the bone marrow niche regulates lymphoblastic leukemia cell dormancy. Blood. (2013) 121(24):4821–31. 10.1182/blood-2012-12-475483 23589674 PMC3682335

[120] Di Tullio A Rouault-Pierre K Abarrategi A Mian S Grey W Gribben J. The combination of CHK1 inhibitor with G-CSF overrides cytarabine resistance in human acute myeloid leukemia. Nat Commun. (2017) 8(1):1679. 10.1038/s41467-017-01834-4 29162833 PMC5698422

[121] Marlow R Honeth G Lombardi S Cariati M Hessey S Pipili A. A novel model of dormancy for bone metastatic breast cancer cells. Cancer Res. (2013) 73(23):6886–99. 10.1158/0008-5472.Can-13-0991 24145351

[122] Vera-Ramirez L Vodnala SK Nini R Hunter KW Green JE. Autophagy promotes the survival of dormant breast cancer cells and metastatic tumour recurrence. Nat Commun. (2018) 9(1):1944. 10.1038/s41467-018-04070-6 29789598 PMC5964069

[123] Guo F Zong S Zhang X Ren Z Shao H Li J. Ferroptosis and metastasis: molecular checkpoints, microenvironmental dynamics, and therapeutic opportunities. Mole Cancer. (2026) 25(1):45. 10.1186/s12943-025-02544-y PMC12947519 41535945

[124] Puente-Cobacho B Esteo C Altea-Manzano P Garcia-Perez JL Quiles JL Sanchez-Rovira P. *De novo* lipogenesis protects dormant breast cancer cells from ferroptosis and promotes metastasis. Redox Biol. (2025) 80:103480. 10.1016/j.redox.2024.103480 39787900 PMC11764609

[125] Lee J Roh JL. Targeting GPX4 in human cancer: implications of ferroptosis induction for tackling cancer resilience. Cancer Lett. (2023) 559:216119. 10.1016/j.canlet.2023.216119 36893895

